# Access before literacy: reframing conversational AI in health self-management for low-resource contexts

**DOI:** 10.3389/ijph.2026.1610302

**Published:** 2026-10-08

**Authors:** Ahmed Saad, Joshua S. Yudkin

**Affiliations:** 1 Department of Business Management, Faculty of Economics and Administration, Masarykova Univerzita, Brno, Czechia; 2 Department of Business Administration, Faculty of Commerce, Benha University, Banha, Egypt; 3 Texas A&M, College Station, TX, United States

**Keywords:** artificial intelligence (AI), health disparities, healthcare access, prompt literacy, self-management support

## Introduction

Qama’s [1] editorial on health self-management with conversational artificial intelligence (AI) makes a compelling case that the usefulness of tools such as ChatGPT for chronic condition management depends on prompt literacy: a user’s ability to frame effective queries and critically interpret AI outputs. The editorial frames prompt literacy as an extension of digital health literacy and a source of disparity: users who prompt effectively can shape a plan suited to their situation, whereas weaker prompting tends to return advice too generic, or too inaccurate, to act on.

This is a valuable observation, but it rests on an assumption that limits its applicability: that the user has access to a healthcare system as an alternative or complement to AI. Where this assumption does not hold, the disparity that matters most may not be a gap in skill but a gap in access. This condition arises across a range of low-resource settings, including low- and middle-income countries (LMICs), as well as under-resourced populations within high-income countries (HICs).

## The access and literacy distinction

Prompt literacy, as Qama [1] defines it, addresses what may be understood as a second-order inequality. It concerns differences among users who have a choice between AI and professional care. It asks how well a person navigates AI as one option within a broader information ecosystem. This fits much of the empirical literature. Alon and Levkovich’s [2] scoping review of 27 studies on generative AI and health information seeking found that credibility and trust concerns were the most commonly reported barriers to AI use, appearing in 48.1% of included studies. The same review found AI was typically part of a broader information routine rather than a replacement for other sources. Similarly, patients with low back pain used AI-generated information but sought professional oversight as perceived stakes increased [3]. These patterns fit a prompt-literacy-style framework because they describe populations for whom professional care remains reachable.

A first-order inequality is different. It concerns whether any accessible guidance exists at all. Where professional care is unaffordable, distant, or otherwise unavailable, the comparison is not AI versus a clinician but AI versus nothing. Under that comparison, even an imperfect AI response may represent an improvement over the counterfactual. Framing AI as preferable to no guidance should not be mistaken for endorsing AI as a substitute for health-system investment. Rather, it highlights the need to evaluate AI tools under the actual counterfactual conditions users face: *rather than displacing prompt literacy, access constraints alter its function.* In settings where AI is used as a substitute rather than a supplement, the consequences of low prompt literacy are amplified, but the appropriate intervention cannot rely only on user training (Table 1).

**TABLE 1:** Access constraints change the meaning of prompt literacy in Artificial Intelligence-supported self-management (Czech Republic, 2026).

Baseline care access	Role of artificial intelligence	Dominant disparity	Main policy concern	Primary intervention logic
Professional care reachable	Artificial intelligence as supplement	Prompt literacy gap	Some users obtain more useful artificial intelligence guidance than others	User training, prompt literacy, critical appraisal
Professional care unreachable or unaffordable	Artificial intelligence as substitute or fallback	Access gap	Artificial intelligence may be the only available source of guidance	Safer default design, escalation guidance, low-resource delivery infrastructure
Mixed or intermittent access	Artificial intelligence as bridge	Access and literacy interact	Users may rely on artificial intelligence while waiting for care	Combine prompt literacy with referral pathways and red-flag detection

## Evidence from the current literature

The evidence on AI-mediated health information seeking is itself geographically narrow. In Alon and Levkovich’s [2] review, the largest shares were conducted in the United States (n = 8), Saudi Arabia (n = 3), the United Kingdom (n = 3), and China (n = 3). The authors explicitly note “a concentration of evidence from English-speaking and high-income settings, with limited representation from low- and middle-income countries, sub-Saharan Africa, Latin America, and South and Southeast Asia.” Only one study was conducted in an African country: Adeyemi et al.'s [4] study in Nigeria. The study’s own authors caution that their convenience sample of university students, recruited online, likely overrepresented those with greater internet access and digital literacy.

This matters not only for representativeness but also for theory. In under-resourced populations within high-income countries, several studies suggest that cost, convenience, stigma, and reduced social friction – not literacy alone – shape AI use. Ayo-Ajibola et al. [5] found that U.S. ChatGPT users were more likely than nonusers to report lower income and lower educational attainment and also scored lower on a validated eHealth literacy scale. Other studies find that chatbots may reduce embarrassment when users seek information about sensitive symptoms, an effect most pronounced for embarrassing sexual symptoms specifically [3, 6]. Yet acceptance cannot be assumed: McLeod et al. [7] found that underserved UK participants valued immediacy and privacy in online support but often rejected chatbots specifically, citing scripted responses and a preference for a live human. Studies with older Black adults further show that credibility, cultural tailoring, and historic mistrust shape responses to health chatbot design [8, 9]. Together, this literature suggests that access-adjacent and structural factors, not literacy, explain most of this pattern.

## When AI is not a supplement

If access rather than skill is the binding constraint for many users, the policy problem changes. The harm attributed to low prompt literacy in an access-constrained setting is not only the harm of using AI less effectively than a skilled peer. It is also the harm of having only one channel of guidance, used imperfectly. These are not equivalent problems. The former can be addressed primarily through user training. The latter requires improving what the AI channel offers by default, including uncertainty signaling, red-flag escalation, and safer design for users with no route to verification.

This distinction also matters for equity. Qama’s [1] argument assumes a shared floor of care access below which literacy differences operate. Where that floor does not exist – because of geography, cost, and/or local health system capacity – the more consequential inequality may be binary rather than graded: whether a person has any accessible source of guidance at all. Treating both situations as the same literacy gap risks under-weighting the more severe access disparity.

The same issue applies to intervention design. Prompt literacy workshops embedded in rehabilitation curricula or delivered through existing care programs presuppose contact with a healthcare system. Where that contact is scarce, these interventions may fail to reach the people who need them. An access-sensitive intervention agenda should therefore supplement, and in some settings precede, user-facing prompt literacy training. Safety cannot depend solely on the user’s ability to ask the right question; it must also be built into the tool and its surrounding delivery infrastructure (Table 2).

**TABLE 2:** Elements of an access-sensitive agenda for conversational Artificial Intelligence in health self-management (Czech Republic, 2026).

Domain	Access-sensitive strategies
Tool design	Uncertainty signaling; red-flag symptom escalation; plain-language outputs; multilingual and low-literacy interfaces; voice-based options
Technical infrastructure	Low-bandwidth design; offline-compatible features where feasible; transparent data and privacy protections
Clinical localization	Alignment with local guidelines; context-specific medication and referral warnings; adaptation to local service availability
Community delivery	Partnerships with community health workers, health clinics, hospitals, pharmacies, schools, universities, libraries, non-government organizations, and public health agencies
Referral pathways	Escalation guidance to available services, including emergency care, clinics, hotlines, or community-based supports
Evaluation measures	Baseline care access; affordability; appointment availability; travel time; language concordance; device and internet access; artificial intelligence used before, instead of, or after professional care

## Toward an access-sensitive research agenda

Alon and Levkovich [2] call for research on generative AI health information seeking in “higher-stakes and underserved contexts, where verification burdens, literacy demands, and access constraints may be especially consequential.” We endorse and extend this call. Access constraint should not be treated as one barrier among many, but as a variable that changes the meaning of outcomes already being measured. Intention to use, verification behavior, and downstream escalation to a clinician mean something different for someone who can see a doctor tomorrow than for someone who cannot see a doctor at all.

Future research should therefore stratify AI adoption and reliance by baseline access, including insurance status, affordability, geographic distance, provider-patient concordance, and whether AI was used before, instead of, or after professional care. Research should also evaluate which access-sensitive design and delivery strategies reduce harm, improve appropriate escalation, and narrow disparities in real-world self-management contexts.

### Conclusion

Prompt literacy remains a useful construct. Better-formed queries can produce more useful outputs, and critical interpretation remains essential. Our claim is narrower: *prompt literacy describes a second-order inequality among users who have a baseline choice, while access describes a first-order inequality over whether that choice exists.* Extending Qama’s [1] disparities argument to populations across both LMICs and HICs for whom that first-order question is not settled requires treating access, not only literacy, as a primary axis of analysis.
